# Differences across males and females in cognitive aging and mortality risk: a trajectory-based approach

**DOI:** 10.1007/s11357-026-02145-7

**Published:** 2026-02-14

**Authors:** Elena Lobo, Concepción De la Cámara, Patricia Gracia-García, Raúl López-Antón, Pedro Saz, David Vaquero-Puyuelo, Antonio Lobo

**Affiliations:** 1 Department of Preventive Medicine and Public Health, Universidad de Zaragoza, C/Domingo Miral S/N, 50009 Zaragoza, Spain; 2 Instituto de Investigación Sanitaria Aragón (IIS Aragón), 50009 Zaragoza, Spain; 3 Centro de Investigación Biomédica en Red de Salud Mental (CIBERSAM), Ministry of Science and Innovation, 28029 Madrid, Spain; 4 Department of Medicine and Psychiatry, Universidad de Zaragoza, 50009 Zaragoza, Spain; 5 Psychiatry Service, Hospital Clínico Universitario, 50009 Zaragoza, Spain; 6 Psychiatry Service, Hospital Universitario Miguel Servet, 50009 Zaragoza, Spain; 7 Department of Sociology and Psychology, Universidad de Zaragoza, 50001 Zaragoza, Spain

**Keywords:** Sex differences, Death risk, Cognitive trajectories, Community sample, ZARADEMP study

## Abstract

This study examined hypothesized differences across males and females in the relationship between cognitive trajectories and mortality in older adults. Data were drawn from the ZARADEMP study on dementia and depression in adults aged 55 and older in Zaragoza, Spain. A total of 2403 cognitively healthy individuals who completed at least three of four waves over a 12-year follow-up were included. Cognitive trajectories were derived from Mini-Mental State Examination (MMSE) scores. Using growth mixture modeling (GMM), four trajectories in men and three in women were previously identified. Mortality data, obtained from official records, covered up to 6 years after the final wave. Cox proportional hazards models assessed mortality risk across trajectories, adjusting for sociodemographic, medical, and lifestyle factors. Mortality was higher among participants with steeper cognitive decline in both sexes. In men, those in the declining-low and stable-medium trajectories had 133% and 36% higher mortality risk, respectively, compared to the stable-high group. In women, declining-low and stable-medium classes had 133% and 30% increased risk, respectively. The association in women followed a clear gradient; in men, this pattern was absent. Differences in mortality predictors were identified: medical conditions (e.g., hypertension, diabetes, alcohol use) were significant only in men, whereas psychosocial and functional variables (e.g., anxiety, ADL dependency, marital status) were significant only in women. This is the first report showing differences across males and females in the association between cognitive decline trajectories and mortality. Sex-specific strategies may be needed to mitigate mortality associated with trajectories of cognitive decline.

## Introduction

Cognitive impairment has been consistently associated with an elevated risk of mortality across diverse population-based studies [1]. Likewise, individuals diagnosed with mild cognitive impairment (MCI) exhibit a significantly increased mortality risk compared to cognitively intact peers. This relationship has been documented in various cohorts, encompassing both community-based populations and clinical samples [2–7].

The relationship between cognitive decline and mortality is complex. Most of the existing research has been limited by a cross-sectional approach, failing to capture the dynamic changes in cognitive function throughout the lifespan [8]. The study of cognitive trajectories across the aging process by using longitudinal data provides a more holistic view of how cognition naturally evolves with age, underscoring the importance of capturing the dynamic and multifaceted nature of cognitive changes, as well as the individual variability in these trajectories [9–11]. Only a small number of studies have explored the long-term impact of cognitive trajectories on future mortality among the oldest age groups. These studies suggest an association between cognitive decline and mortality, and their findings reflect the heterogeneity of the aging process, as different mortality risks have been observed across the various identified trajectories [8, 12–15].

We have previously emphasized the importance of investigating the influence of sex on cognitive trajectories [16], aligning with the broader objective “to bring sex and gender into the mainstream of modern medical research” [17]. The impact of sex on cognitive decline remains a subject of ongoing controversy [18–20] and warrants further clarification [21]. With regard to mortality, emerging evidence indicates sex-related differences in the association between cognitive impairment and survival. A recent study involving American adults aged 60 years and older, based on data from the National Health and Nutrition Examination Survey, found that lower cognitive performance was associated with increased mortality risk, with stronger associations observed among women and individuals with lower educational attainment [1]. Similarly, a systematic review by Lin et al. focusing on the general population aged 60 years and above reported that women with baseline cognitive impairment exhibited a higher risk of all-cause mortality compared to men with equivalent cognitive impairment, even after adjusting for sociodemographic and health-related variables [22]. Although both studies underscore the role of sex in modulating mortality risk, neither explicitly examined cognitive trajectories over time. Duan et al. assessed cognitive decline (as measured by MMSE) and all-cause mortality at different time points in a cohort of the oldest-old [23]. They reported that cognitive decline was associated with an increased risk in all-cause mortality, the risk being significantly higher in women and individuals with hypertension. However, to date, no study has independently explored within-sex variability in cognitive trajectories and their association with mortality.

Therefore, there remains significant potential for further investigation into the differences by sex in the relationship between cognitive trajectories and mortality. Moreover, we have not found information related to predictors of mortality in cognitive trajectories independently in men and women. This type of research is relevant in view of diverse predictors reported in different cognitive trajectories [16], which may have clinical and preventive implications. In this background, this study was intended to test the following hypotheses:

- There is a higher mortality for declining cognitive trajectories in both men and women.
- The association of declining cognitive trajectories and mortality will be different in women and in men.
- Differences across the male and female samples in mortality predictors will also be observed.

## Methods

This sub-study is part of the ZARADEMP project, a longitudinal, population-based investigation focused on dementia and depression in adults aged 55 and older in Zaragoza, the fifth most populous city in Spain. A random, representative sample, stratified by age and sex with proportional allocation, including institutionalized individuals, was recruited and followed over a period of twelve years in four waves. A comprehensive description of the general methodology can be found elsewhere [24].

A total of 4803 individuals were included, which consisted of a participation rate of 79.5%. A two-phase case finding for dementia in each wave was used. Dementia and “subsyndromal” dementia cases at baseline, identified with the Geriatric Mental State (GMS), with its cognitive section and its Automated Geriatric Examination for Computer Assisted Taxonomy package (AGECAT) criteria [25], were excluded in the follow-up.

Written informed consent, privacy, and confidentiality have been maintained throughout the Project according to the Helsinki Convention principles, and the Ethics Committee of the University of Zaragoza and the Fondo de Investigación Sanitaria (FIS) approved the study (CP16/2012, 19 September 2012), following the Spanish Law.

### Assessment of cognitive function

Validated, Spanish versions of the GMS and AGECAT and the Mini-Mental State Examination, MMSE, were used to assess orientation in time and space, memory, attention, calculation, language, and visuoperception [26, 27].

Each wave of the study consisted of two phases. In phase 1, cognitive data was obtained in the elderly’s home by senior medical students specially trained as lay interviewers who administered the instruments. This information was thoroughly reviewed by the research psychiatrists supervising the interviewers individually. Two months later, in phase 2, and blind to the results of phase 1, the probable cases of dementia were re-examined by the research psychiatrists.

Trajectories of change over time in cognitive function had been modeled with data from the MMSE administered in phase 1 using growth mixture models (GMM) separately for men and women [16]. Only participants with at least three measurements of MMSE were included. The technique considers different baseline ages and number of time points for classifying participants into clusters with similar trajectories according to their longitudinal data of MMSE at each wave, assuming that individual differences in trajectories can be summarized by a finite set of different polynomial functions for age or time [28, 29]. We have previously reported the results of this analysis: a 4-class growth model for men was the best fitting age-adjusted model, which comprised classes 1-declining low, 2-declining high, 3-stable high, and 4-stable medium cognitive function over time, with 13.5%, 4.8%, 53%, and 28.8% of the men sample, respectively [16]. In women, a 3-class growth model was the best fitting age-adjusted model that included trajectories 1-declining, 2-stable medium, and 3-stable high that classified women respectively with 2.4%, 47.4%, and 50.2% of them [16]. The trajectories identified are now used in this study.

### Confounders

Information on sex, age, marital status, education, and lifestyle including alcohol and tobacco consumption was collected in the baseline interview. Education was categorized as no studies, basic (elementary school, complete or incomplete), and high (high school and/or university). Activities of daily living, basic (bADLs), and instrumental (iADLs), were assessed by disability scales, Katz’s index, and Lawton and Brody scale, respectively, included in the History and Aetiology Schedule (HAS). Data on diabetes and hypertension was obtained through the European Studies of Dementia (EURODEM) Risk Factors Questionnaire [30]. Information on depression and anxiety syndromes was provided by the GMS interview and the respective sections in the AGECAT computer system. After symptom assessment (AGECAT Stage 1), the diagnosis of both depression and anxiety emerge from stage 2. In this stage, a computer program compares syndrome clusters (e.g., dementia, depression, anxiety) to reach a final diagnosis, recorded as either a diagnostic “subsyndromal” (confidence levels 1 and 2) or a diagnostic “case” (confidence levels 3). The “cases” level was used in this particular study.

### Ascertainment of mortality

The techniques for identifying deaths in the ZARADEMP project have been previously reported [7]. The determination of all-cause mortality of each participant was based on the official population registry in the city. Death certificates, which included accurate information on the day, month, and year of death, were used to complete and verify the information. Time was calculated for each subject as days from baseline assessment to the date of death, and those individuals remaining alive or missing (emigrated, not localizable) 6 years after the fourth wave were considered censored.

### Data analysis

All data analyses were stratified by sex and are interpreted descriptively. Baseline characteristics were described by mean and standard deviation (SD) for continuous variables and proportions for categorical. Chi-squared and Student’s *t* test tests were used to compare differences. In order to analyze associations between cognitive trajectories (classes 1 to 3) and time to death during follow-up, as well as to analyze the predictors of mortality within each trajectory, Cox proportional hazards regression models were used, estimating hazard ratios (HR) with 95% confidence intervals (CI). The proportional hazards (PH) assumption was assessed in the Cox models using Schoenfeld residual analysis, employing both global and covariate-specific tests. No significant violations of the assumption were found for cognitive trajectories or for the covariates included in the fitted models. Subjects not registered as dead were considered censored at the last date when follow-up information was assessed, or lost to follow-up, whichever occurred first. Cox models were adjusted for a set of covariates selected a priori based on previous evidence of their clinical and epidemiological relevance as potential confounders in the association between cognitive trajectories and mortality. Age, baseline MMSE score, education level, marital status, hypertension, diabetes, depression, anxiety, dependence in basic (bADLs) and instrumental (iADLs) activities of daily living, alcohol consumption, and tobacco use were selected because they showed relevant associations with life status in the descriptive analyses and because of their known relationship with cognitive decline and all-cause mortality. While the analyses were stratified by sex, the same set of adjustment variables was found to be associated with the exposure and outcome variables in each strata, and, therefore, they were included in the adjusted models for both men and women. SPSS software v.26 [31] was used for all the analyses.

## Results

From the 1061 men and 1342 women included in the present analyses and after 18 years from baseline, 49.6% (526) and 36.8% (494) had died at follow-up, respectively. As shown in Table 1, at baseline and compared with those alive/censored, men and women who died were older, more frequently widowed or single. For both men and women, the proportions of hypertension, depression, and dependency in ADLs were higher, and the mean MMSE score at baseline was lower among the deceased. In men, the frequency of those who had basic or no studies and of ex-drinkers at baseline was higher for those who died. Compared with alive women, those who died had more frequently basic studies.

**Table 1:** Baseline characteristics of MEN and WOMEN participants, by survival status

	Men (***N*** = 1061)			Women (***N*** = 1342)		
	Death cases^b^ ***n*** (%)/mean (SD)	Survivors/censored^c^ ***n*** (%)/mean (SD)	***p***-value	Death cases^d^ ***n*** (%)/mean (SD)	Survivors/censored^e^ ***n*** (%)/mean (SD)	***p***-value
Age	73.4 (8.3)	66.3 (5.8)	< 0.001	76 (7.8)	67.5 (6.1)	< 0.001
High education level						
No studies	28 (5.4)	16 (3)		59 (12)	48 (5.7)	
Basic	369 (69.7)	365 (68.5)	0.045	375 (76.1)	664 (78.7)	< 0.001
High	123 (23.7)	152 (28.5)		59 (12)	132 (15.6)	
Marital status						
Widowed	64 (12.2)	24 (4.5)		233 (47.2)	202 (23.8)	< 0.001
Single^a^	37 (7)	33 (6.2)	< 0.001	86 (17.4)	95 (11.2)	
Couple	425 (80.8)	478 (89.3)		175 (35.4)	551 (65)	
Hypertension	360 (68.7)	308 (57.6)	< 0.001	384 (77.7)	558 (65.9)	< 0.001
Diabetes	69 (13.2)	52 (9.8)	0.079	68 (13.9)	93 (11)	0.126
Depression	39 (7.6)	22 (4.2)	0.019	135 (29)	183 (22.3)	0.008
Anxiety	12 (2.3)	6 (1.1)	0.144	35 (7.1)	42 (5)	0.105
bADLs dependency	26 (5)	11 (2.1)	0.010	40 (8.1)	25 (3)	< 0.001
iADLs dependency	41 (7.8)	19 (3.6)	0.003	88 (17.8)	34 (4)	< 0.001
Alcohol						
Abstainer	162 (30.8)	176 (33)	< 0.001	416 (84.2)	699 (82.4)	
Occasional	29 (5.5)	47 (8.8)		10 (2)	30 (3.5)	0.439
Habitual	211 (40)	236 (44.3)		46 (9.3)	84 (9.9)	
Ex-drinker	124 (23.6)	74 (13.9)		22 (4.5)	35 (4.1)	
Smoking status			0.111			
Non-smoker	138 (26.2)	164 (30.7)		462 (93.5)	783 (92.3)	0.418
Smoker and ex-smoker	388 (73.8)	371 (69.3)		32 (6.5)	65 (7.7)	
MMSE at baseline	27.4 (2.4)	28.2 (2)	< 0.001	26.8 (2.4)	27.8 (2)	< 0.001
Cognitive trajectories						
Class 1: decliners low	95 (18.1)	48 (9)		22 (4.5)	10 (1.2)	
Class 2: decliners high	16 (3)	35 (6.5)	< 0.001	256 (51.8)	380 (44.8)	< 0.001
Class 3: stable high	121 (23)	184 (34.4)		216 (43.7)	458 (54)	
Class 4: stable medium	294 (55.9)	268 (50.1)				

Note. *iADLS* instrumental activities of daily living, *bADLs* basic activities of daily living, *MMSE* mini-mental status examination

^a^Single, separated

^b^*N* = 526

^c^*N* = 535

^d^*N* = 494

^e^*N* = 848

Compared to alive men, those who died during follow-up had been classified more frequently in classes 1 and 4, and less frequently in classes 2 and 3. Compared to alive women, those who died during follow-up had been classified more frequently in classes 1 and 2, and less frequently in class 3.

**Fig. 1:**
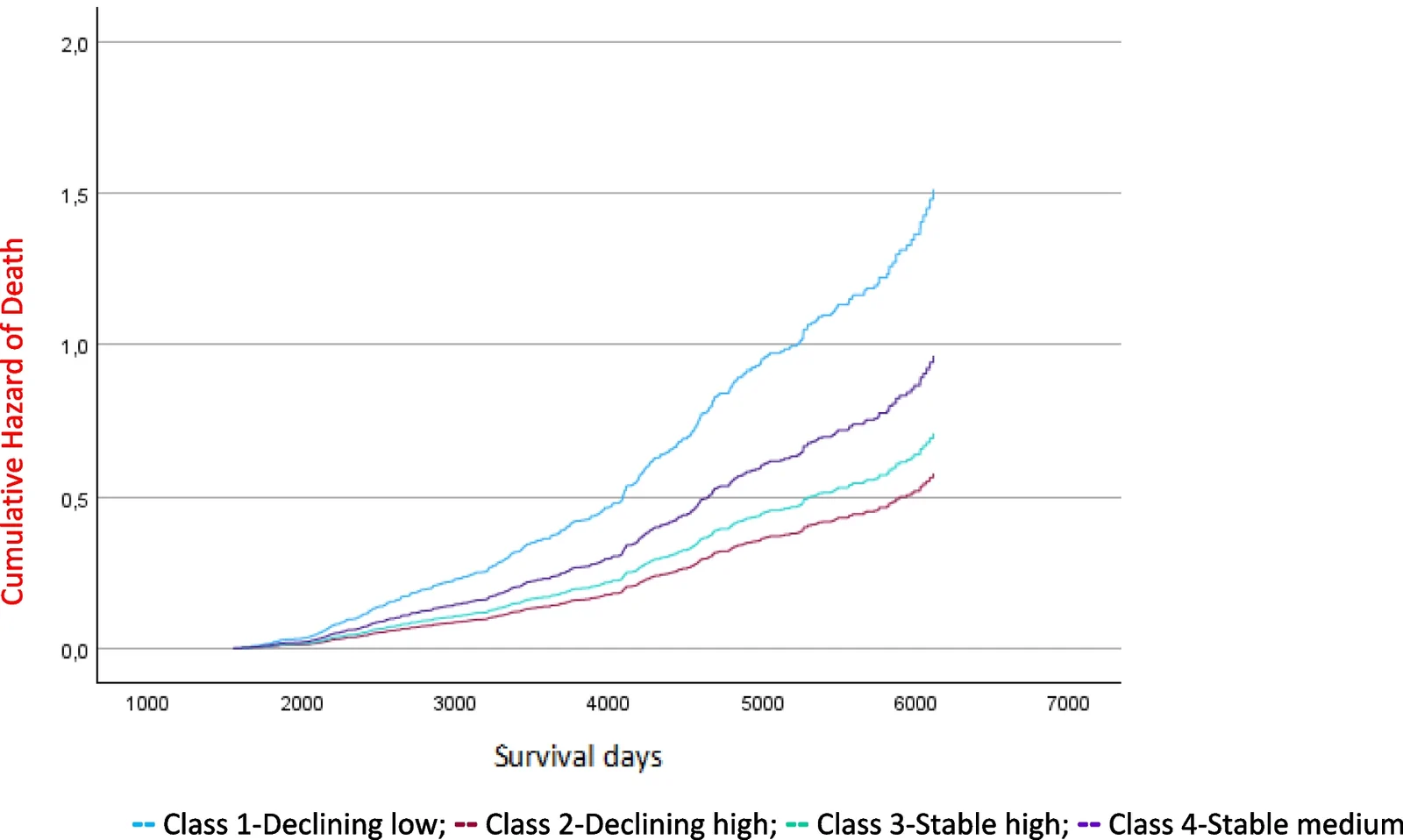
Cumulative hazard function of death for Cognitive Trajectories in men. Adjusted cox regression hazard function in relation to death for men, calculated in days. The hazard of death is significantly increased in men in class-1-declining-low (light blue) compared to the other three classes. Men in class 4-stable-medium (purple) had increased hazard than those in classes 3 (green) and 2 (red), but was only significant compared to the hazard risk of those in class 3

Among men, the Cox proportional hazards regression analyses showed different associations of the cognitive trajectories with the risk of death after controlling for potential confounders. Figure 1 shows the cumulative hazard function (curve) for each cognitive trajectory. The average time to death was 9.9 years (± 3.2). Hazard of death increased through follow-up time, but the amount of risk was different for each moment and class. Table 2 shows that, compared with class-3-stable high, men grouped in classes 1-declining low and 4-stable medium had a 133% (HR = 2.33; 95% CI = 1.53–2.97; *p* < 0.001) and 36% (HR = 1.36; 95% CI = 1.07–1.72; *p* = 0.011) higher risk of mortality, respectively. Class 2-declining high was not associated in the adjusted model (HR = 0.81; 95% CI = 0.48–1.38; *p* = 0.444). Compared with class 4-stable medium, those individuals in class 1-declining low had a 57% (HR = 1.57; 95% CI = 1.21–2.05; *p* < 0.001) higher risk of death, and no statistical differences were seen with class 2-declining high (HR = 0.60; 95% CI = 0.35–1.01; *p* = 0.056) (Not in tables). Compared with class 2-declining high, those men in class 1 had 162% higher risk of mortality (HR = 2.62; 95% CI = 1.47–4.69; *p* = 0.001) (not in tables).

**Table 2:** Hazard ratios (95% CI) for the association between cognitive trajectories and risk of mortality, crude and adjusted for potential confounders, in men

	Crude					Adjusted*			
	HR	95% CI			***p***-value	HR	95% CI		***p***-value
Age	1.11	1.10	1.12		< 0.001	1.11	1.10	1.13	< 0.001
Baseline MMSE score	0.92	0.90	0.94		< 0.001	1.00	1.00	1.10	0.973
Hypertension	1.42	1.20	1.71		< 0.001	1.26	1.04	1.53	0.021
Diabetes	1.27	0.98	1.63		0.069	1.32	1.01	1.74	0.045
Depression	1.66	1.20	2.30		0.002	1.26	0.90	1.78	0.178
Anxiety	1.93	1.09	3.42		0.024	1.65	0.89	3.05	0.111
iADLs dependency	2.52	1.64	3.10		< 0.001	1.03	0.71	1.49	0.875
bADLs dependency	2.29	1.54	3.40		< 0.001	1.44	0.93	2.24	0.104
Alcohol intake (Ref. abstainer)									
Occasional	0.76	0.51	1.13		0.169	0.77	0.51	1.16	0.209
Habitual	1.00	0.81	1.22		0.971	1.03	0.83	1.28	0.774
Ex-drinker	1.54	1.22	1.94		< 0.001	1.36	1.06	1.74	0.017
Civil status (Ref. widow)									
Single^a^	0.54	0.36	0.82		0.003	1.11	0.71	1.73	0.653
Coupled	0.45	0.35	0.59		< 0.001	1.02	0.76	1.37	0.899
Ex-/smoker	1.16	0.96	1.41		0.126	1.32	1.07	1.62	0.010
Education (Ref. high)									
No studies	2.02	1.34	3.05		< 0.001	1.17	0.73	1.86	0.524
Basic	1.20	.98	1.47		0.079	0.93	0.74	1.16	0.513
Cognitive trajectories (Ref. class 3-stable high)									
Class 1-declining low	2.06	1.57	2.70	< 0.001		2.33	1.53	2.97	< 0.001
Class 2-declining high	0.68	0.40	1.14	0.144		0.81	0.48	1.38	0.444
Class 4-stable medium	1.49	1.20	1.84	< 0.001		1.36	1.07	1.72	0.011

Note. *iADLS* instrumental activities of daily living, *bADLs* basic activities of daily living, *MMSE* mini-mental status examination

^*^Adjusted by: age, baseline MMSE score, education level, marital status, hypertension, diabetes, depression, anxiety, dependence in basic (bADLs) and instrumental (iADLs) activities of daily living, alcohol consumption, and tobacco use

^a^Single, separated

Referring covariates, age, hypertension, diabetes, alcohol ex-drinkers, and tobacco smokers/ex-smokers were associated with an increased risk of death.

**Fig. 2:**
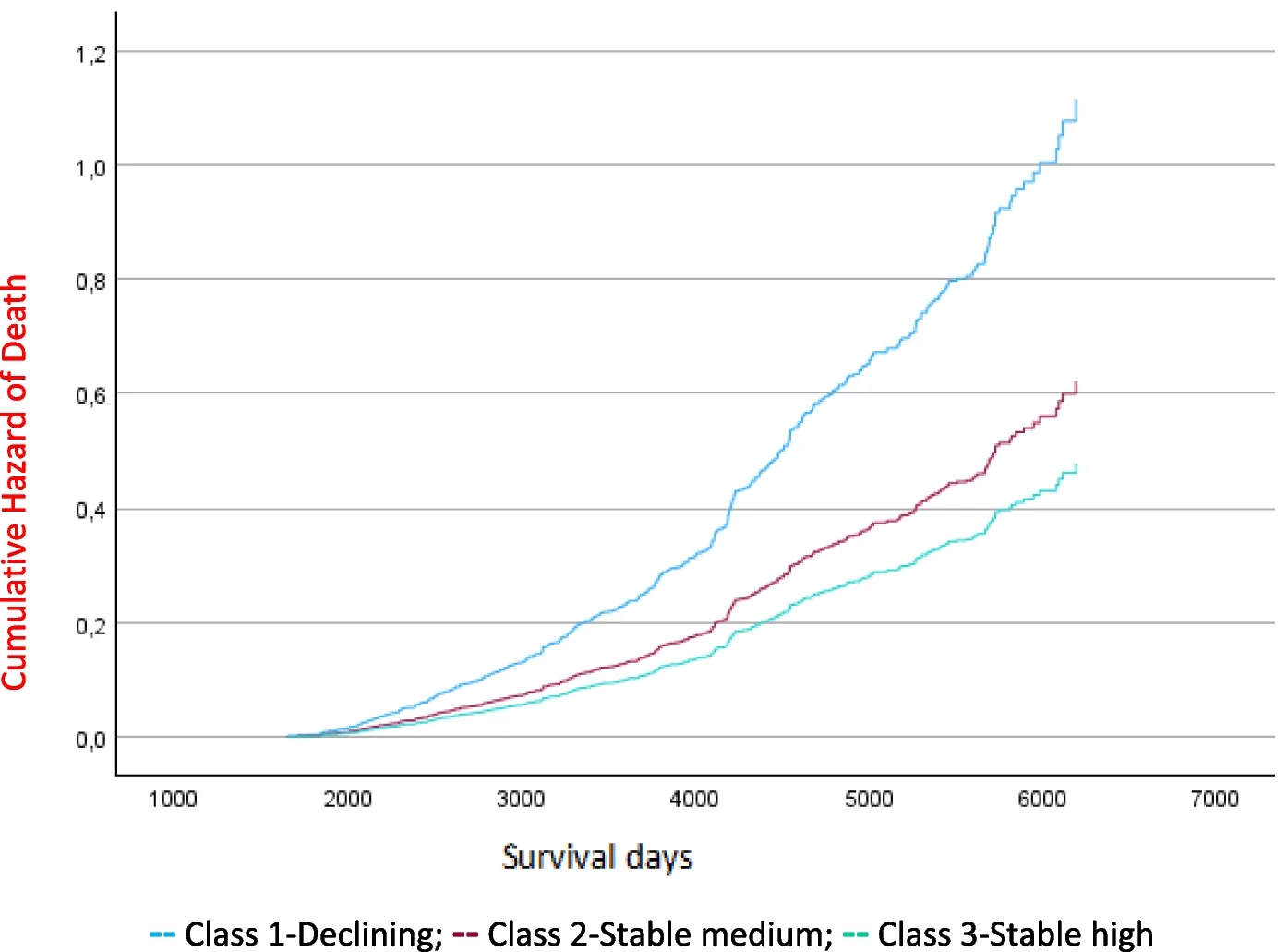
Cumulative hazard function of death for Cognitive Trajectories in women. Adjusted cox regression hazard function in relation to death for women, calculated in days. The hazard of death is significantly increased in women in class-1-declining (light blue) compared to the other two classes. Women in class 2-stable-medium (red) had also increased hazard of death than those in class 3-stable-high (green)

Among women, the results of the adjusted Cox proportional hazards regression analyses showed a gradient in the association of the cognitive trajectory with the risk of death as represented in the cumulative hazard function (curve) for each cognitive trajectory in Fig. 2. The average time to death was 10.2 years (± 3), being 10, 10.2, and 10.3 years for classes 1, 2, and 3, respectively. Women grouped in class 1-declining low and in class 2-stable medium had a 133% (HR = 2.33, 95% CI (1.40–3.90), *p* = 0.001) and 30% (HR = 1.30, 95% CI (1.04–1.63), *p* = 0.024) higher risk of death, respectively, than women in class 3-stable high (Table 3). When comparing classes 2 and 1, those in class 1 had 80% higher risk of death (HR = 1.80, 95% CI (1.10–2.93), *p* = 0.020) (not in tables).

**Table 3:** Hazard ratios (95% CI) for the association between cognitive trajectories and risk of mortality, crude and adjusted for potential confounders, in women

	Crude				Adjusted*			
	HR	95% CI		***p***-value	HR	95% CI		***p***-value
Age	1.14	1.13	1.16	< 0.001	1.125	1.11	1.14	< 0.001
Baseline MMSE score	0.90	0.83	0.90	< 0.001	0.981	0.93	1.03	0.442
Hypertension	1.68	1.35	2.10	< 0.001	1.08	0.90	1.36	0.496
Diabetes	1.22	0.94	1.58	0.128	1.24	0.94	1.63	0.131
Depression	1.30	1.06	1.59	0.010	1.11	0.90	1.36	0.352
Anxiety	1.35	0.96	1.91	0.085	1.80	1.25	2.59	0.002
iADLs dependency	3.52	2.79	4.44	< 0.001	1.43	1.08	1.89	0.013
bADLs dependency	2.54	1.84	3.51	< 0.001	1.37	0.94	2.00	0.099
Alcohol intake (Ref. abstainer)								
Occasional	0.63	0.33	1.17	0.143	0.97	0.50	1.90	0.927
Habitual	0.90	0.67	1.23	0.515	0.86	0.62	1.19	0.361
Exdrinker	1.10	0.72	1.69	0.665	1.14	0.73	1.78	0.563
Civil status (Ref. widow)								
Single^a^	0.82	0.64	1.5	0.110	1.20	0.92	1.56	0.186
Coupled	0.35	0.29	.42	< 0.001	0.74	0.59	0.92	0.008
Ex-/smoker	0.87	0.61	1.25	0.462	1.50	1.02	2.19	0.038
Education (Ref. high)								
No studies	2.90	1.46	3.00	< 0.001	0.93	0.61	1.41	0.715
Basic	1.20	0.92	1.58	0.186	0.84	0.62	1.14	0.266
Cognitive trajectories (Ref. class 3-stable high)								
Class 1: declining	2.74	1.77	4.26	< 0.001	2.33	1.40	3.87	0.001
Class 2: stable medium	1.33	1.11	1.60	0.002	1.30	1.04	1.63	0.024

Note. *iADLS* instrumental activities of daily living, *bADLs* basic activities of daily living, *MMSE* mini-mental status examination

^*^Adjusted by: age, baseline MMSE score, education level, marital status, hypertension, diabetes, depression, anxiety, dependence in basic (bADLs) and instrumental (iADLs) activities of daily living, alcohol consumption, and tobacco use

^a^Single, separated

Referring covariates, age, anxiety, dependency in iADLs, and tobacco smokers/ex-smokers were associated with an increased risk of death; on the contrary, being coupled was associated with lower death risk.

## Discussion

To our knowledge, this is the first study analyzing separately for men and women the association of cognitive trajectories and mortality. We have found some support for the hypotheses tested. First, the mortality in both men and women was in general higher in the cognitive trajectories showing higher decline. Specifically, in men, compared with class-3-stable high, individuals in classes 1-declining low and 4-stable medium had a 133% and 36% higher risk of mortality, respectively. In women, those in class 1-declining low and in class 2-stable medium had a 133% and 30% higher risk of death, respectively, than women in class 3-stable high. Our present study also confirms the heterogeneity in the aging process, since different mortality risks were documented for the diverse trajectories identified.

Second, different patterns of association between cognitive decline and mortality were observed across the male and female subsamples. In women, the association was consistent and followed a gradient pattern since the mortality risk was stronger in both trajectories with cognitive decline (class 1 and class 2) when compared to women with high and stable functioning through the years (class 3); and the risk was higher in class 1, the one with poorest cognitive performance and with rapid decline, when compared with class 2, the one maintaining an intermediate level of performance. The pattern of the association between cognitive decline and mortality was different in men, as it was not so consistent, and a gradient was not observed: the most declining trajectory (class 1-declining low) had higher death risks than the rest of the trajectories but, at the same time, the risk for class 2-declining high was not different when compared to the stable groups, classes 3 and 4*.*

Intriguingly, men in class 2 showed initially high cognitive performance and a declining trajectory through aging, but ultimately exhibited the lowest mortality rate among all classes: it was 31.4% in class 2 compared to 66.4%, 39.7%, and 52.3% in classes 1, 3, and 4, respectively (*p* < 0.001). This suggests a protective trend, which reached statistical significance only when compared to class 1 (declining–low) in the multivariate survival analyses. This observation may be interpreted through the lens of the cognitive reserve (CR) hypothesis. It has been shown that individuals with higher CR not only perform more efficiently at baseline but also experience a more gradual cognitive decline relative to those with lower CR [32]. Notably, higher CR has also been associated with reduced mortality risk [33]. Although our study lacks a comprehensive assessment of CR, our data reveal that men in class 2 had the highest baseline global cognitive performance and had attained significantly higher levels of education (37.3%) compared to individuals in class 1 (8.4%) and class 4 (17.6%), and similar to those in class 3 (47.5%) [16]. Educational attainment is widely recognized as a primary contributor to CR and, independently, has been linked to lower adult mortality rates [34].

Finally, in support of the third hypothesis, differences across the male and female subsamples were also observed related to mortality predictors. While higher age and tobacco use determined higher death risk in both men and women, clinical and behavioral circumstances such as hypertension, diabetes, and alcohol intake (ex-drinkers) were only associated in men, and psychosocial and functional variables such as anxiety, dependency in ADLs, and civil status were associated only in women.

The available information related to differences between men and women in the association between cognitive trajectories and mortality is very limited. Furthermore, it is difficult to compare our results with previous research, since the methods implemented are different. Most previous research has identified cognitive trajectories for global samples, not independently for men and women [8, 13, 15, 35]. All these studies were conducted in other cultures and used similar cognition assessments and analogous procedures for modeling the trajectories. All of them found associations between trajectories of cognitive decline and mortality, but the follow-up was shorter in all and sex was only included as a confounding factor in the analysis of the association. We similarly reported the association in a previous study in the same sample and female sex, included in the analysis of confounding factors, was found to be associated with a lower risk of death [12].

Other studies reporting the association of cognitive decline and mortality have used very different methodologies, such as the Yaffe et al. report, that included only women in their sample [36]. Duan et al., in the study of oldest old individuals referred to in the introduction, observed that female sex, low blood pressure, and the absence of hypertension positively affected the cognitive decline–mortality association. However, they used sex interaction in the analysis of the association between cognitive decline and mortality, rather than modeling cognitive trajectories using complex techniques such as GMM, and no independent analysis of men and women was conducted [23]. In the systematic review by Lin et al. focusing on the general population aged 60 or more years, it was concluded that women with cognitive impairment at baseline had to face an 8% higher risk of all-cause mortality than men with similar cognitive impairment [22]. However, neither the cognitive trajectories over time nor within-sex variability in cognitive trajectories were explicitly examined.

Other two relevant studies used a trajectory-based approach. Zhao et al. investigated a large sample of individuals aged 65 years or above in the Chinese Longitudinal Healthy Longevity Survey [37]. They discussed that “gender disparity in cognitive decline was noteworthy, with a significantly lower proportion of males in both moderate and rapid decline groups.” Walsh et al. conducted a study of cognitive trajectories in the United States, although their objective was different, “to better understand the temporal dynamics of progression from cognitive decline to onset of dementia” [38]. They documented that the rate of change in cognitive trajectories predicted the risk of mortality above and beyond the initial cognitive level. The effect of sex was highly significant in both different models, the women having a hazard ratio (HR) of 0.67 and 0.56 in respect to men. However, they only analyzed “non-dementia mortality,” contrary to our study on all-cause mortality, their sample size was much smaller than ours, and they simultaneously modeled longitudinal cognitive change and time-to-dementia/death processes. Furthermore, none of these two studies modeled cognitive trajectories independently for men and for women, and neither analyzed separately the mortality risk in men and in women.

There is limited evidence to explain the observed differences across the male and female subsamples in mortality associated with cognitive trajectories, although several hypotheses might be proposed. Among biological explanations, genetic risk factors must be considered, including mechanisms related to sex-specific vulnerabilities such as age-related X chromosome inactivation skewing, alterations in the gut microbiome, and maternally inherited mitochondrial DNA variants [19]. The neuroprotective potential of estrogen in mitigating cognitive decline and its associated risks has been reported [39]. Moreover, low testosterone levels in older women may exert detrimental, domain-specific effects on cognitive performance [40]. Sex-based differences in susceptibility to various diseases [19], as well as in the presence of comorbid conditions that elevate mortality risk, such as cardiovascular disease [23] must also be taken into account.

Psychosocial factors must also be pondered. As shown in the systematic review by Ferreira et al., between the ages of 60 and 80, contextual and cultural influences affecting men and women may contribute to differing rates of cognitive decline between the sexes [20]. A nationwide US cohort study further showed that more frequent leisure-time physical activity is associated with better cognitive function [41]. Traditional lifestyle risk factors in men such as smoking, alcohol consumption, and physical inactivity have been linked to their excess mortality compared to women [42]. Conversely, women may be more susceptible to the adverse effects of psychological distress on mortality [43].

This study strengthens the evidence linking trajectories of cognitive decline with increased mortality risk, reporting differences across the male and female subsamples for the first time and highlighting associations that may surpass those of traditional risk factors such as poor health status or depressive symptoms. The findings underscore the critical importance of routine cognitive screening and longitudinal monitoring in older adults, aiming to facilitate early detection and inform personalized interventions that promote healthy life expectancy. Notably, sex-specific patterns emerged: in women, a clear gradient of risk was observed, reinforcing the need for consistent early detection and timely preventive measures. In contrast, the less defined association in men may reflect a moderating effect of cognitive reserve. Additionally, distinct modifiable risk factors were identified: hypertension, diabetes, and alcohol consumption in men; and anxiety and dependency in activities of daily living in women, underscoring the relevance of tailored preventive strategies. Overall, these results contribute to the growing body of literature advocating for sex-sensitive, longitudinal approaches to cognitive aging and mortality, offering valuable insights for the design of targeted public health interventions.

Among other strengths of this research, a unique contribution of the study is the independent analysis for men and women of the association of cognitive trajectories and mortality. Moreover, the study examined a large and representative population sample and used repeated measures data that allowed the identification, with GMM methodology, of cognitive trajectories during follow-up. The observation of mortality 6 years after the identification of the cognitive trajectories provides robustness to our results.

There are limitations to be acknowledged, including those related to response rate, longitudinal dropout, or the possibility of a survivor bias because only participants with 3 or more MMSE measurements were included in GMM. The present study recruited, with a very stringent procedure, individuals who were cognitively healthy at baseline, therefore impeding the observation of the risk of mortality of trajectories in individuals with cognitive deficits at baseline. The study might have failed to detect a relationship between cognitive decline and mortality due to other competing causes of death, although the analyses were adjusted for multiple factors of incident disease. Medication usage or certain environmental factors were not controlled. While our study was conducted among a population in a rather typical city in Spain, our findings might not be applicable to populations of different ethnicities or socioeconomic backgrounds.

## Conclusions

The association between cognitive decline trajectories and mortality was confirmed in this study, with differences across the male and female samples identified for the first time. Specifically, the association was more consistent and followed a gradient pattern only among women. Furthermore, the predictors of mortality differed across men and women samples: hypertension, diabetes, and alcohol consumption were associated with mortality only in men, whereas psychosocial and functional factors, including anxiety, dependency in activities of daily living (ADLs), and marital status, were associated only in women. These findings underscore not only the importance of early detection and longitudinal monitoring of cognitive function in the aging population but also the need for sex-specific strategies to mitigate mortality associated with cognitive decline. Additionally, this study represents a significant contribution to guide ongoing research on aging and the development of interventions aimed at increasing healthy life expectancy.

## Data Availability

The data presented in this study are available upon reasonable request to the corresponding author.

## References

[1] Adjoian Mezzaca T, Dodds L V., Rundek T, Zeki Al Hazzouri A, Caunca MR, Gomes-Osman J, et al. Associations between cognitive functioning and mortality in a population-based sample of older united states adults: differences by sex and education. J Aging Health [Internet]. 2022 [cited 2025 May 7];34:905–15. Available from: https://pubmed.ncbi.nlm.nih.gov/35440227/. 10.1177/08982643221076690 35440227

[2] Dewey ME, Saz P. Dementia, cognitive impairment and mortality in persons aged 65 and over living in the community: a systematic review of the literature. Int J Geriatr Psychiatry. 2001;16:751–61. 10.1002/gps.397 11536341

[3] An R, Liu GG. Cognitive impairment and mortality among the oldest-old Chinese. Int J Geriatr Psychiatry. 2016;31:1345–53. 10.1002/gps.4442 26891153

[4] Bae J Bin, Han JW, Kwak KP, Kim BJ, Kim SG, Kim JL, et al. Impact of mild cognitive impairment on mortality and cause of death in the elderly. J Alzheimer’s Dis [Internet]. 2018 [cited 2025 May 6];64:607–16. Available from: https://pubmed.ncbi.nlm.nih.gov/29914024/. 10.3233/JAD-171182 29914024

[5] Sachs GA, Carter R, Holtz LR, Smith F, Stump TE, Tu W, et al. Cognitive impairment: an independent predictor of excess mortality: a cohort study.[Summary for patients in Ann Intern Med. 2011 Sep 6;155(5):I34; PMID: 21893619]. Ann Intern Med [Internet]. 2011 [cited 2025 May 7];155:300–8. Available from: http://ovidsp.ovid.com/ovidweb.cgi?T=JS&CSC=Y&NEWS=N&PAGE=fulltext&D=med7&AN=21893623. 10.7326/0003-4819-155-5-201109060-00007 21893623

[6] Fogg C, Meredith P, Culliford D, Bridges J, Spice C, Griffiths P. Cognitive impairment is independently associated with mortality, extended hospital stays and early readmission of older people with emergency hospital admissions: a retrospective cohort study. Int J Nurs Stud. 2019;96:1–8. 10.1016/j.ijnurstu.2019.02.005 30850127

[7] Santabárbara J, Lopez-Anton R, Marcos G, De-La-Cámara C, Lobo E, Saz P, et al. Degree of cognitive impairment and mortality: a 17-year follow-up in a community study. Epidemiol Psychiatr Sci. 2015. 10.1017/S2045796014000390. PMC8367364 24905936

[8] Wei Y, Zhang Y, Li Y, Meng F, Zhang R, You Z, et al. Trajectories of cognitive change and their association with all-cause mortality among Chinese older adults: results from the Chinese longitudinal healthy longevity survey. Behav Sci (Basel, Switzerland) [Internet]. 2025 [cited 2025 May 6];15(3):365. Available from: https://www.ncbi.nlm.nih.gov/pubmed/40150260. 10.3390/bs15030365 PMC11939546 40150260

[9] Lobo E, Gracia-García P, Lobo A. Longitudinal trajectories of cognitive aging. Curr Opin Psychiatry [Internet]. 2024 [cited 2024 Jun 5];37:123–9. Available from: https://pubmed.ncbi.nlm.nih.gov/38226551/. 10.1097/YCO.0000000000000918 38226551

[10] Salthouse TA. Selective review of cognitive aging. J Int Neuropsychol Soc [Internet]. 2010 [cited 2024 Jun 5];16:754–60. Available from: https://pubmed.ncbi.nlm.nih.gov/20673381/. 10.1017/S1355617710000706 PMC3637655 20673381

[11] Wu Z, Phyo AZZ, Al-harbi T, Woods RL, Ryan J. Distinct cognitive trajectories in late life and associated predictors and outcomes: a systematic review. J Alzheimers Dis Rep. 2020;4:459–78. 10.3233/ADR-200232 PMC7683100 33283167

[12] Lobo E, De la Cámara C, Gracia-García P, Saz P, López-Antón R, Lobo A. Cognitive trajectories in older adults and associated mortality and predictors. Soc Psychiatry Psychiatr Epidemiol [Internet]. 2025 [cited 2025 May 15]; Available from: https://www.ncbi.nlm.nih.gov/pubmed/40047850. 10.1007/s00127-025-02862-y PMC12594731 40047850

[13] Wu Z, Woods RL, Chong TTJ, Orchard SG, Shah RC, Wolfe R, et al. Cognitive trajectories in community-dwelling older adults and incident dementia, disability and death: a 10-year longitudinal study. Front Med [Internet]. 2022 [cited 2023 Jul 20];9:917254. Available from: https://www.ncbi.nlm.nih.gov/pubmed/35833102. 10.3389/fmed.2022.917254 PMC9271785 35833102

[14] Hu X, Gu S, Sun X, Gu Y, Zhen X, Li Y, et al. Cognitive ageing trajectories and mortality of Chinese oldest-old. Arch Gerontol Geriatr. 2019;82:81–7. 10.1016/j.archger.2019.01.018 PMC6451875 30716682

[15] Taniguchi Y, Kitamura A, Ishizaki T, Fujiwara Y, Shinozaki T, Seino S, et al. Association of trajectories of cognitive function with cause-specific mortality and medical and long-term care costs. Geriatr Gerontol Int. 2019;19:1236–42. 10.1111/ggi.13802 31746115

[16] Lobo E, Lobo A, Gracia-García P, López-Antón R, Saz P, De la Cámara C. Sex differences in longitudinal trajectories of cognitive aging in Zaragoza, Spain. Am J Geriatr Psychiatry [Internet]. 2023 [cited 2023 Jul 20]; Available from: https://www.ncbi.nlm.nih.gov/pubmed/37169710. 10.1016/j.jagp.2023.04.012 37169710

[17] Mauvais-Jarvis F, Bairey Merz N, Barnes PJ, Brinton RD, Carrero JJ, DeMeo DL, et al. Sex and gender: modifiers of health, disease, and medicine. Lancet (London, England) [Internet]. 2020 [cited 2022 Sep 22];396:565–82. Available from: https://pubmed.ncbi.nlm.nih.gov/32828189/. 10.1016/S0140-6736(20)31561-0 PMC7440877 32828189

[18] McCarrey AC, An Y, Kitner-Triolo MH, Ferrucci L, Resnick SM. Sex differences in cognitive trajectories in clinically normal older adults. Psychol Aging [Internet]. 2016 [cited 2021 Apr 21];31:166–75. Available from: https://pubmed.ncbi.nlm.nih.gov/26796792/. 10.1037/pag0000070 PMC4783196 26796792

[19] Ostan R, Monti D, Gueresi P, Bussolotto M, Franceschi C, Baggio G. Gender, aging and longevity in humans: an update of an intriguing/neglected scenario paving the way to a gender-specific medicine. Clin Sci (Lond) [Internet]. 2016 [cited 2022 Sep 22];130:1711–25. Available from: https://pubmed.ncbi.nlm.nih.gov/27555614/. 10.1042/CS20160004 PMC4994139 27555614

[20] Ferreira L, Ferreira Santos-Galduróz R, Ferri C, Fernandes Galduróz J. Rate of cognitive decline in relation to sex after 60 years-of-age: a systematic review. Geriatr Gerontol Int [Internet]. 2014 [cited 2021 Sep 30];14:23–31. Available from: https://pubmed.ncbi.nlm.nih.gov/23682773/. 10.1111/ggi.12093 23682773

[21] Bloomberg M, Dugravot A, Dumurgier J, Kivimaki M, Fayosse A, Steptoe A, et al. Sex differences and the role of education in cognitive ageing: analysis of two UK-based prospective cohort studies. Lancet Public Heal. 2021;6:e106–15. 10.1016/S2468-2667(20)30258-9 PMC8141610 33516287

[22] Lin S, Chen M. Gender-specific impact of cognitive impairment on all-cause mortality in older persons: a meta-analysis. Exp Gerontol. 2022. 10.1016/j.exger.2022.111860. 35640780

[23] Duan J, Sam NB, Wang SJ, Liu Y. Exploring the association between cognitive decline and all-cause mortality with blood pressure as a potential modifier in oldest old individuals. Sci Rep. 2022. 10.1038/s41598-022-21487-8. PMC9556626 36224279

[24] Lobo A, Saz P, Marcos G, Día J-L, De-La-Cámara C, Ventura T, et al. The ZARADEMP Project on the incidence, prevalence and risk factors of dementia (and depression) in the elderly community: II. Methods and first results. Eur. J. Psychiat. [online] 2005, vol. 19, n.1, pp.40-54. Available from: https://scielo.isciii.es/scielo.php?script=sci_arttext&pid=S0213-61632005000100004&lng=es.

[25] Lobo A, Saz P, Marcos G, Día JL, De-la Cámara C. The prevalence of dementia and depression in the elderly community in a southern European population: the Zaragoza study. Arch Gen Psychiatry [Internet]. 1995 [cited 2021 May 20];52:497–506. Available from: https://jamanetwork.com/journals/jamapsychiatry/fullarticle/497118. 10.1001/archpsyc.1995.03950180083011 7771920

[26] Folstein MF, Folstein SE, McHugh PR. Mini-Mental-State”, a practical method for grading the cognitive state of patients for the clinician. J Psychiatr Res. 1975;12:189–98. 10.1016/0022-3956(75)90026-6 1202204

[27] Lobo A, Saz P, Marcos G, Día JL, de la Cámara C, Ventura T, et al. Revalidation and standardization of the cognition mini-exam (first Spanish version of the Mini-Mental Status Examination) in the general geriatric population. Med Clin (Barc). 1999;112:767–74. 10422057

[28] Nagin DS. Analyzing developmental trajectories: a semiparametric, group-based approach. Psychol Methods [Internet]. 1999 [cited 2021 May 20];4:139–157. Available from: 10.1037/1082-989X.4.2.139.

[29] Jung T, Wickrama KAS. An introduction to latent class growth analysis and growth mixture modeling. Soc Personal Psychol Compass. 2008;2:302–17.

[30] Launer LJ, Brayne C, Breteler MM. Epidemiologic approach to the study of dementing diseases: a nested case-control study in European incidence studies of dementia. Neuroepidemiology. 1992;11:114–8. 10.1159/000111005 1603240

[31] IBM Corp. IBM SPSS Statistics for Windows, Version 26.0. Armonk, NY: IBM Corp; 2019. Available from: https://www.ibm.com/products/spss.

[32] Montemurro S, Rumiati RI, Pucci V, Nucci M, Mondini S. Cognitive reserve can impact trajectories in ageing: a longitudinal study. Aging Clin Exp Res [Internet]. 2025 [cited 2025 May 6];37:93. Available from: https://www.ncbi.nlm.nih.gov/pubmed/40095141. 10.1007/s40520-025-03000-z PMC11914246 40095141

[33] Zijlmans JL, Lamballais S, Lahousse L, Vernooij MW, Ikram MK, Ikram MA, et al. The interaction of cognitive and brain reserve with frailty in the association with mortality: an observational cohort study. Lancet Heal Longev [Internet]. 2021 [cited 2025 May 15];2:e194–201. Available from: https://pubmed.ncbi.nlm.nih.gov/36098120/. 10.1016/S2666-7568(21)00028-3 36098120

[34] Balaj M, Henson CA, Aronsson A, Aravkin A, Beck K, Degail C, et al. Effects of education on adult mortality: a global systematic review and meta-analysis. Lancet Public Heal [Internet]. 2024 [cited 2025 Jul 8];9:e155–65. Available from: https://pubmed.ncbi.nlm.nih.gov/38278172/. 10.1016/S2468-2667(23)00306-7 PMC10901745 38278172

[35] Zang E, Zhang Y, Wang Y, Wu B, Fried TR, Becher RD, et al. Association between cognitive trajectories and subsequent health status, depressive symptoms, and mortality among older adults in the United States: findings from a nationally-representative study. J Gerontol A Biol Sci Med Sci. 2024. 10.1093/gerona/glae143. PMC11212484 38845419

[36] Yaffe K, Peltz CB, Ewing SK, McCulloch CE, Cummings SR, Cauley JA, et al. Long-term cognitive trajectories and mortality in older women. J Gerontol A Biol Sci Med Sci. 2016;71:1074–80. 10.1093/gerona/glw003 PMC4945886 26843186

[37] Zhao Y, Zhou W, Xing M, Zhang L, Tong Y, Lv X, et al. Later-life cognitive trajectories and risk of death: results from a 6-year longitudinal study of 7082 Chinese. J Prev Alzheimers Dis. 2024. 10.14283/jpad.2024.96. 39350388

[38] Walsh CE, Yang YC, Oi K, Aiello A, Belsky D, Harris KM, et al. Age profiles of cognitive decline and dementia in late life in the aging, demographics, and memory study. J Gerontol B Psychol Sci Soc Sci. 2022. 10.1093/geronb/gbac038. PMC9535777 35171992

[39] Gurvich C, Hoy K, Thomas N, Kulkarni J. Sex differences and the influence of sex hormones on cognition through adulthood and the aging process. Brain Sci [Internet]. 2018 [cited 2025 Jun 19];8:163. Available from: https://pmc.ncbi.nlm.nih.gov/articles/PMC6162653/. 10.3390/brainsci8090163 PMC6162653 30154388

[40] Dratva MA, Banks SJ, Panizzon MS, Galasko D, Sundermann EE. Low testosterone levels relate to poorer cognitive function in women in an APOE-ε4-dependant manner. Biol Sex Differ 2024 151 [Internet]. 2024 [cited 2025 Jul 8];15:1–15. Available from: 10.1186/s13293-024-00620-4. PMC11151480 38835072

[41] Lee DH, Rezende LFM, Joh HK, Keum N, Ferrari G, Rey-Lopez JP, et al. Long-term leisure-time physical activity intensity and all-cause and cause-specific mortality: a prospective cohort of US adults. Circulation [Internet]. 2022 [cited 2025 Jul 8];146:523–34. Available from: 10.1161/CIRCULATIONAHA.121.058162. PMC9378548 35876019

[42] Wu YT, Niubo AS, Daskalopoulou C, Moreno-Agostino D, Stefler D, Bobak M, et al. Sex differences in mortality: results from a population-based study of 12 longitudinal cohorts. CMAJ [Internet]. 2021 [cited 2025 May 28];193:E361–70. Available from: https://pmc.ncbi.nlm.nih.gov/articles/PMC8096404/. 10.1503/cmaj.200484 PMC8096404 33722827

[43] Mendes De Leon CF, Krumholz HM, Seeman TS, Vaccarino V, Williams CS, Kasl S V., et al. Depression and risk of coronary heart disease in elderly men and women: new Haven EPESE, 1982–1991. Arch Intern Med [Internet]. 1998 [cited 2025 May 28];158:2341–8. Available from: https://pubmed.ncbi.nlm.nih.gov/9827785/. 10.1001/archinte.158.21.2341 9827785

